# Low-Latency Edge-Enabled Digital Twin System for Multi-Robot Collision Avoidance and Remote Control

**DOI:** 10.3390/s25154666

**Published:** 2025-07-28

**Authors:** Daniel Poul Mtowe, Lika Long, Dong Min Kim

**Affiliations:** 1 Department of ICT Convergence, Graduate School, Soonchunhyang University, Asan 31538, Republic of Korea; 2 Department of Internet of Things, Soonchunhyang University, Asan 31538, Republic of Korea

**Keywords:** digital twin, edge computing, low latency, networked control system, collision avoidance

## Abstract

This paper proposes a low-latency and scalable architecture for Edge-Enabled Digital Twin networked control systems (E-DTNCS) aimed at multi-robot collision avoidance and remote control in dynamic and latency-sensitive environments. Traditional approaches, which rely on centralized cloud processing or direct sensor-to-controller communication, are inherently limited by excessive network latency, bandwidth bottlenecks, and a lack of predictive decision-making, thus constraining their effectiveness in real-time multi-agent systems. To overcome these limitations, we propose a novel framework that seamlessly integrates edge computing with digital twin (DT) technology. By performing localized preprocessing at the edge, the system extracts semantically rich features from raw sensor data streams, reducing the transmission overhead of the original data. This shift from raw data to feature-based communication significantly alleviates network congestion and enhances system responsiveness. The DT layer leverages these extracted features to maintain high-fidelity synchronization with physical robots and to execute predictive models for proactive collision avoidance. To empirically validate the framework, a real-world testbed was developed, and extensive experiments were conducted with multiple mobile robots. The results revealed a substantial reduction in collision rates when DT was deployed, and further improvements were observed with E-DTNCS integration due to significantly reduced latency. These findings confirm the system’s enhanced responsiveness and its effectiveness in handling real-time control tasks. The proposed framework demonstrates the potential of combining edge intelligence with DT-driven control in advancing the reliability, scalability, and real-time performance of multi-robot systems for industrial automation and mission-critical cyber-physical applications.

## 1. Introduction

Multi-robot systems are increasingly deployed in various domains such as industrial automation, warehouse logistics, search and rescue, and autonomous navigation [1]. These systems require efficient coordination mechanisms to ensure smooth operation while avoiding collisions in dynamic environments [2]. Traditional collision avoidance methods predominantly rely on onboard sensors, such as Light Detection and Ranging (LiDAR) sensors and cameras, to detect obstacles and adjust trajectories in real time. While effective in certain scenarios, sensor-based approaches face several limitations, including occlusion issues, environmental noise, and limited predictive capabilities [3]. Moreover, real-time onboard processing of sensor data imposes computational burdens, often leading to delays in trajectory adjustments and suboptimal collision avoidance [4].

Remote-controlled multi-robot systems have also been widely adopted, allowing human operators to intervene and guide robots [5]. However, these systems are constrained by high communication overhead and latency, particularly when transmitting a large volume of raw sensor data such as video streams. The reliance on high-bandwidth communication links introduces network congestion and increased response times, which significantly impact the effectiveness of collision avoidance in large-scale deployments. To address these challenges, integrating DT technology with remote-controlled multi-robot systems presents a promising approach. DT creates a virtual replica of a physical system, enabling real-time monitoring, predictive analytics, and simulation-based optimization. Through continuous synchronization between the physical and digital counterparts, DT-based systems can improve trajectory planning and proactive collision avoidance, mitigating the limitations of conventional sensor-based and purely remote-controlled strategies. However, existing DT-based network control systems face challenges in communication efficiency, data synchronization, and real-time responsiveness due to the high volume of data transmission required to maintain accurate digital representations [6].

This research proposes an Edge-Enabled Digital Twin networked control system (E-DTNCS) to enhance multi-robot collision avoidance and remote control by leveraging edge computing. The primary novelty of this work lies not in the individual technologies of Digital Twins (DTs) or edge computing, but in their integration into a cohesive architecture that strategically decouples perception from control. A core principle of this architecture is this separation of concerns: the Edge Layer is responsible for performing semantic feature extraction to ensure communication efficiency (i.e., latency reduction) [7], which in turn enables the Digital Twin Layer to provide high-level control intelligence (i.e., predictive collision avoidance). By offloading perception and solving the data transmission bottleneck inherent in traditional DTNCS, our framework provides the low-latency data stream necessary to make the DT’s predictive capabilities viable for real-time, proactive safety, a critical gap that has limited their practical application in dynamic multi-agent systems.

The primary objective of this research is to design, develop, and empirically validate an E-DTNCS architecture for multi-robot collision avoidance and remote control in wireless networked environments. The proposed framework seeks to overcome the critical limitations associated with conventional sensor-based and direct remote-control systems, such as high communication latency, bandwidth saturation, and reactive-only decision-making. By integrating edge computing, DT models, feature-based communication, and a guarding-circle-based collision avoidance mechanism, this work aims to establish a scalable and latency-resilient control architecture for next-generation industrial automation and collaborative robotics.

**Figure 1:**
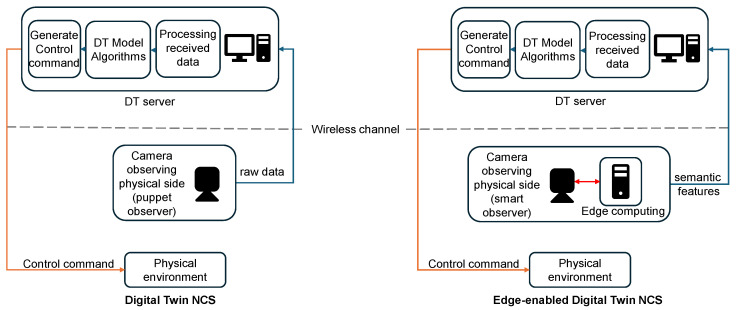
Brief block diagram of the conventional DTNCS and the proposed E-DTNCS.

To illustrate the novelty of our approach, Figure 1 summarizes the contributions of our research by comparing the conventional digital twin networked control systems (DTNCSs) with the proposed E-DTNCS. Conventional DTNCSs rely heavily on transmitting raw sensor data, leading to excessive communication overhead, high latency, and synchronization challenges. In contrast, our proposed E-DTNCS integrates edge computing for localized data preprocessing and feature-based communication, significantly reducing the communication burden while ensuring near-instantaneous synchronization and enabling proactive, low-latency collision avoidance.

The key contributions of this paper are as follows:

1. A novel DT-based multi-robot collision avoidance system that integrates real-time predictive modeling and trajectory optimization to enhance navigation accuracy.
2. An edge computing-based smart observer architecture that preprocesses and extracts critical motion data before transmission, significantly reducing bandwidth usage compared to conventional raw sensor data transmission.
3. A communication-efficient feature-based data transmission model that replaces raw video feeds with compressed trajectory data, achieving lower latency and improved real-time responsiveness.
4. An implementation of an E-DTNCS that demonstrates seamless real-world synchronization between physical robots and their digital counterparts.
5. Extensive experimental validation comparing the proposed system with conventional sensor-based and remote-controlled approaches, demonstrating significant improvements in collision avoidance accuracy and latency reduction.

The remainder of this paper is organized as follows. Section 2 reviews related works in collision avoidance and digital twin systems. Section 3 presents the system model, including the architecture and a formal analysis of network latency and its impact on safety. Section 4 details the proposed E-DTNCS framework and the guarding circle algorithm. Section 5 describes the experimental setup and procedures. Section 6 presents and discusses the results. Section 7 presents limitations and future work. Finally, Section 8 concludes the paper and outlines future work.

## 2. Related Works

Collision avoidance has long been a cornerstone in the development of autonomous robotic systems, with the primary goal of ensuring safe and efficient navigation in dynamic and often unpredictable environments. Traditional approaches to collision avoidance heavily rely on onboard sensor systems such as LiDAR, stereo cameras, infrared, and ultrasonic sensors. For instance, LiDAR provides high-precision 3D point clouds, enabling accurate spatial awareness and obstacle detection in cluttered environments [8,9]. Similarly, stereo vision and depth cameras enrich spatial understanding by offering semantic scene interpretation [10,11,12]. However, these methods place significant computational demands on embedded systems and suffer from susceptibility to environmental conditions such as lighting, reflectivity, and occlusion.

To overcome the limitations, sensor fusion techniques have emerged as a robust solution, combining complementary data streams from heterogeneous sensors to improve overall perception accuracy and fault tolerance [13,14]. While sensor fusion enhances performance, it further exacerbates computational overhead, making real-time deployment on resource-constrained platforms challenging.

On the architectural front, centralized monitoring and control systems have been widely adopted, particularly in structured environments like warehouses and smart manufacturing floors [15,16]. These frameworks leverage cloud computing to facilitate global situational awareness, coordinated task scheduling, and inter-robot communication. However, such centralized models introduce critical limitations, including increased communication latency, reduced scalability, and vulnerability to single points of failure. The emergence of edge-computing paradigms has introduced a middle-ground solution that offloads computationally intensive tasks from individual robots to nearby edge servers, thus reducing latency while preserving responsiveness [17].

The DT concept, initially established in the context of cyber-physical systems and Industry 4.0 [18], represents a paradigm shift in system modeling and real-time monitoring. By creating a digital counterpart of a physical entity, DTs enable real-time synchronization, predictive analytics, and virtual experimentation. In autonomous robotics, DTs are gaining traction for enabling model-based planning and proactive fault detection [19,20,21,22,23,24,25]. Despite this progress, most DT applications in robotics are still limited to simulation environments or offline data analysis, lacking the real-time, closed-loop control necessary for high-risk and dynamic settings like multi-robot navigation.

This study addresses these gaps by proposing a novel E-DTNCS framework that tightly couples the virtual model with the physical robot network. The proposed system introduces a low-latency communication pipeline between a smart observer, an edge server (hosting the DT), and the mobile robots. Unlike previous work, this architecture leverages decision models embedded within the DT environment to perform real-time collision prediction and control signal generation. This approach not only reduces computational strain on individual agents but also enables a scalable, coordinated response to dynamic environmental changes, thereby pushing the boundary of DT utility in autonomous robotics.

## 3. System Model

This section details the theoretical model of the proposed Edge-Enabled Digital Twin Networked Control System (E-DTNCS). We first describe the overall system architecture, then define the state representation of the robots, and finally, we provide a formal analysis of the network communication model, which is critical for ensuring the system’s low-latency performance and safety.

### 3.1. System Architecture

**Figure 2:**
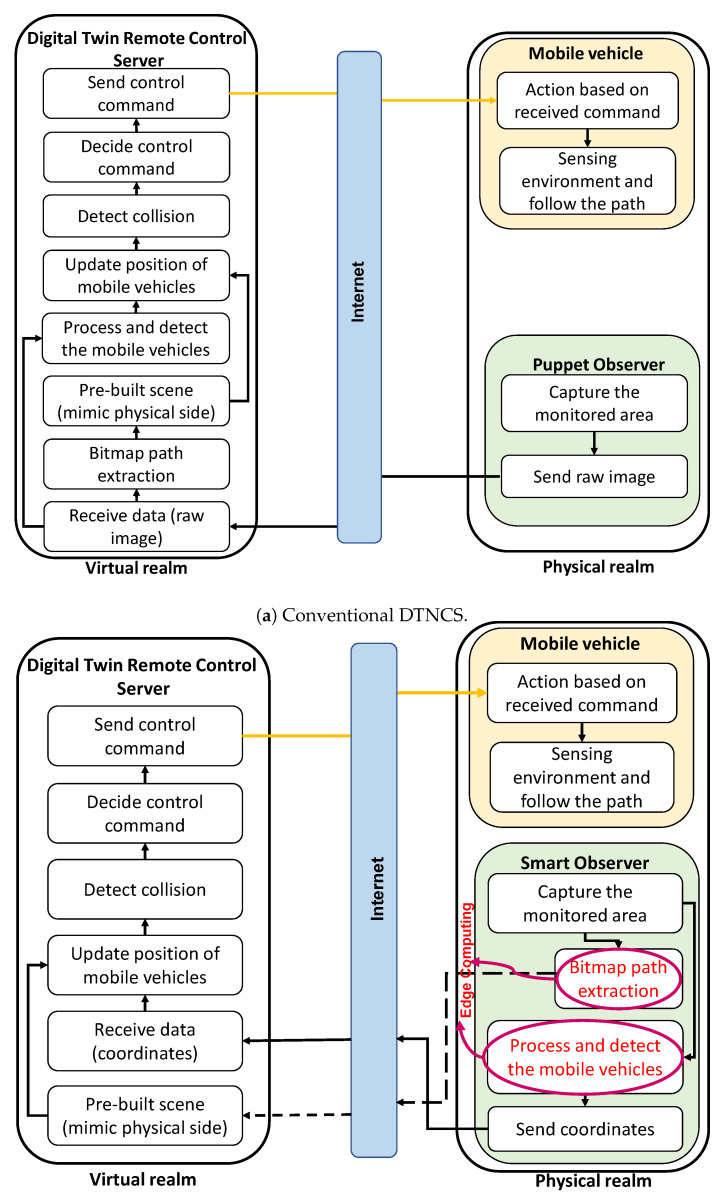
Detailed block diagram of the conventional DTNCS and the proposed E-DTNCS.

The E-DTNCS architecture comprises three main layers operating in a closed loop, as depicted in Figure 2.

- The Physical Layer (Mobile Vehicle): This layer consists of a fleet of *N* mobile robots, denoted by the set R={1,2,…,N}, navigating in a shared physical workspace. Each robot is a simple agent with basic locomotion capabilities and is responsible for executing control commands received from the DT server.
- The Edge Layer (Smart Observer): An off-board sensing unit, such as a camera mounted with a view of the workspace, connected to an edge-computing device (e.g., a single-board computer). This smart observer captures raw sensor data (e.g., video frames) of the entire operational area, performs real-time feature extraction to determine the state of each robot, and transmits this compact feature information to the DT server.
- The Digital Twin Layer (DT Server): A remote server that hosts the digital twin of the entire multi-robot system. It receives feature data from the edge observer, updates the state of the virtual robots in the simulation, runs predictive collision avoidance algorithms, and generates and sends corrective control commands back to the physical robots via a wireless network.

This layered architecture offloads the perception and decision-making tasks from the individual robots to the more powerful edge and server components, allowing the robots to be lightweight and energy-efficient.

Our E-DTNCS implementation adheres to a three-tier hierarchical architecture, comprising the Physical Layer, the Edge Layer, and the Digital Twin Layer. This structure is in accordance with the layered digital twin modeling methodologies [26] and standards such as ISO 23247 [27]. While this structural alignment provides a robust foundation, the primary novelty of our work lies in how these layers are operationalized to solve the dual challenges of communication latency and predictive control in a multi-agent system. This approach enables the system to implement a predict-then-act paradigm, where safety decisions are made not in reaction to collisions, but in anticipation of them, a critical departure from conventional feedback control loops.

### 3.2. Robot State Representation

The state of each physical robot i∈R at any given time *t* is represented by a pose vector $p_{i}\left(t\right)$. For our 2D navigation scenario, this vector includes the robot’s position, orientation, and velocity:

$$\begin{array}{c} p_{i}\left(t\right)={\left[x_{i}\left(t\right),y_{i}\left(t\right),\theta_{i}\left(t\right),v_{i}\left(t\right)\right]}^{T}, \end{array}$$

where $\left(x_{i},y_{i}\right)$ are the Cartesian coordinates of the robot’s center, $\theta_{i}$ is its heading angle, and $v_{i}$ is its forward linear velocity. The digital twin server maintains a virtual representation, or twin, ${\hat{p}}_{i}\left(t\right)$, for each robot, which is continuously updated to mirror the state of its physical counterpart, i.e., ${\hat{p}}_{i}\left(t\right)\approx p_{i}\left(t\right)$.

### 3.3. Network Communication and Low-Latency Requirements

For the E-DTNCS to be effective, the wireless round-trip delay must be sufficiently short to guarantee that the virtual model remains a faithful predictor of the physical robots’ states. This is crucial for proactive collision avoidance.

#### 3.3.1. End-to-End Latency Model

The total end-to-end latency, $t_{e2e}$, is the time elapsed from the moment the smart observer captures the state of the physical system to the moment a physical robot executes a resulting control command. In our E-DTNCS, this is primarily governed by the uplink communication. The downlink packets carrying control commands are very small and their contribution to the latency is considered negligible. The total end-to-end latency is modeled as follows:

(1) $$\begin{array}{c} t_{e2e}\left(P\right)=t_{\mathrm{enc}}+t_{\mathrm{air}}\left(P\right)+t_{\mathrm{queue}}+t_{\mathrm{proc}}, \end{array}$$

where *P* is the size of the uplink data payload in bytes, $t_{\mathrm{enc}}$ is the encoding time at the edge observer, $t_{\mathrm{air}}$ is the over-the-air transmission time (proportional to *P*), $t_{\mathrm{queue}}$ is the queuing and medium-access delay in the wireless network, and $t_{\mathrm{proc}}$ is the processing time at the DT server. Our proposed system drastically reduces *P* by sending features instead of raw data, thereby minimizing $t_{\mathrm{air}}$ and $t_{\mathrm{queue}}$.

#### 3.3.2. Delay-Robust Safety Condition

**Figure 3:**
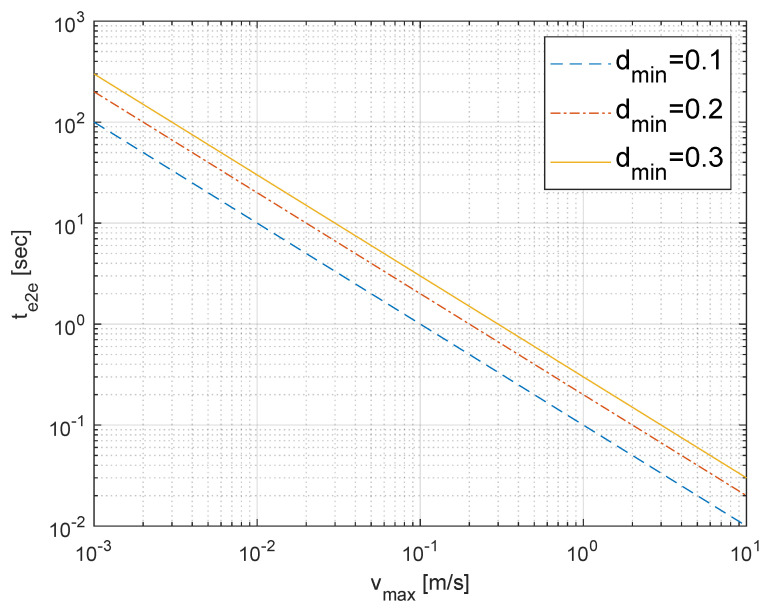
The feasible region for delay-robust safety. For a given minimum safe distance ($d_{\min}$), the system is safe as long as the end-to-end latency ($t_{e2e}$) and maximum robot velocity ($v_{\max}$) fall within the region below the corresponding line.

A fundamental requirement for safe operation is that any corrective action must be taken before a collision becomes unavoidable. This imposes a strict upper bound on the permissible end-to-end latency. If two robots are approaching each other, the system must detect the potential conflict and actuate a response within the time it takes for them to travel a minimum safe distance, $d_{\min}$. This condition can be expressed as follows:

(2) $$\begin{array}{c} t_{e2e}\left(P\right)<\frac{d_{\min}}{v_{\max}}, \end{array}$$

where $v_{\max}$ is the maximum relative velocity of the robots. If the total latency $t_{e2e}$ is less than this time-to-collision threshold, the system can guarantee a response before the safety margin $d_{\min}$ is breached. This inequality defines a *feasible region* for safe operation, as illustrated in Figure 3. For a given safety distance $d_{\min}$, any operating point $\left(v_{\max},t_{e2e}\right)$ that falls below the boundary line is considered safe. The goal of our E-DTNCS design is to minimize $t_{e2e}$ to ensure the system always operates well within this feasible region.

#### 3.3.3. Lyapunov Stability with Time-Evolving Distance Error Bound

To further formalize safety under communication delays, we extend the Lyapunov-based stability analysis to incorporate a time-delayed feedback and derive a time-evolving bound on the inter-robot distance error. Let $d_{ij}\left(t\right)$ be the measured inter-robot distance between neighbor robots *i* and *j* at time *t*. Let $e_{d}\left(t\right)$ denote the distance error; for instance, $e_{d}\left(t\right)$ can be defined as the shortfall in separation between two robots compared to the minimum safe distance, $e_{d}\left(t\right)=d_{ij}\left(t\right)-d_{\min}$, so that $e_{d}\left(t\right)\geq 0$ when the robots are safely apart, and $e_{d}\left(t\right)<0$ would indicate a violation of the safety distance. In a delay-free scenario, a suitable Lyapunov function $V(e_{d})$ (e.g., $V=\frac{1}{2}e_{d}^{2}$) can be used to design a control law that ensures $e_{d}\left(t\right)$ decays to zero over time, maintaining stability and keeping the robots a safe distance apart. Let $\dot{V}≜\frac{d}{dt}V(e_{d}\left(t\right))$ be the time-derivative of the Lyapunov function along the system trajectories. Because $\dot{V}<0$ whenever $e_{d}>0$, the distance error decreases monotonically, remains non-negative for all *t*, and converges asymptotically to zero, thereby guaranteeing collision avoidance.

When the end-to-end delay $t_{e2e}$ is present in the feedback loop, the control action at time *t* is based on stale information from time $t-t_{e2e}$. The closed-loop error dynamics, ${\dot{e}}_{d}\left(t\right)$, can be conceptually written as follows:

(3) $$\begin{array}{c} {\dot{e}}_{d}\left(t\right)=f\left(e_{d}\left(t\right),e_{d}\left(t-t_{e2e}\right)\right), \end{array}$$

for some function *f* determined by the control law and robot dynamics. A common approach is to show that the delayed system remains ultimately stable if $t_{e2e}$ is below a critical value. Using the Lyapunov–Razumikhin method for time-delay systems [28], one can establish an exponential bound on the error. In particular, if the delay condition $t_{e2e}<\frac{d_{\min}}{v_{\max}}$ is satisfied, the distance error will decay exponentially within a bounded envelope. For example, one can derive a bound of the following form:

(4) $$\begin{array}{c} e_{d}\left(t\right)\leq e_{d}\left(0\right)e^{-\lambda \left(t-t_{e2e}\right)},\;t\geq t_{e}2e, \end{array}$$

for some decay rate λ>0, which is related to the Lyapunov function’s convergence rate. This indicates that after an initial lag of at most $t_{e2e}$, the error starts decaying exponentially. Importantly, as long as the initial error $e_{d}\left(0\right)$ does not violate safety (e.g., robots start at a safe distance, so $e_{d}\left(0\right)\geq 0$) and $t_{e2e}$ meets the above bound, $e_{d}\left(t\right)$ will remain non-negative for all t>0. In other words, the robots never get closer than $d_{\min}$. The control system, despite the delay, corrects the spacing fast enough to prevent collision. The time-evolving upper bound on $e_{d}\left(t\right)$ guarantees that the gap deviation caused by a delayed actuation will be corrected before it leads to a collision. Thus, the system is delay-stable in the sense of safety: the inter-robot distance might temporarily decrease when a hazard is detected (since the response is $t_{e2e}$ seconds late), but it will not fall below $d_{\min}$ if the delay is within the safe limit.

If the delay is larger than $d_{\min}/v_{\max}$, this stability assurance breaks down. One can no longer guarantee $e_{d}\left(t\right)\geq 0$. In fact, with excessive delay, the error may become negative (meaning the actual distance dropped below the minimum before correction), which corresponds to a collision. The Lyapunov analysis thus reinforces that $t_{e2e}$ must be bounded to maintain safety. It also provides insight into how the distance error evolves over time; essentially, $e_{d}\left(t\right)$ will follow a delayed exponential decay. There may be an initial error growth during the delay interval, but if $t_{e2e}$ is within the safe bound, that growth is limited by $e_{d}\left(t_{e2e}\right)=e_{d}\left(0\right)$ (in the worst case) and thereafter $e_{d}\left(t\right)$ decays. The result is a bounded tracking error for the inter-robot distance, ensuring the spacing eventually returns to safe levels even after a perturbation or sudden stop of one robot.

## 4. Edge-Enabled Digital Twin Collision Avoidance Framework

Building on the system model, this section describes our proposed E-DTNCS framework for multi-robot collision avoidance. We detail the key components that enable low-latency operation: edge-based feature extraction and the guarding circle algorithm running on the DT server.

### 4.1. Framework Overview

The core novelty of our framework is the strategic division of labor between the edge and the DT server to minimize latency. The workflow is as follows:

1. Sensing: A smart observer with a global view of the workspace captures a video stream of the robots.
2. Edge Feature Extraction: Instead of transmitting the high-bandwidth video stream, the edge device processes each frame locally to extract low-dimensional features, specifically, the (x,y) coordinates of each robot.
3. Feature Transmission: These compact feature packets are transmitted over a wireless network to the DT server. This step significantly reduces the data payload compared to sending raw images, as shown in the results in Section 6.
4. Digital Twin Synchronization: The DT server receives the feature packets and updates the states of the corresponding virtual robots in its simulation environment.
5. Predictive Collision Avoidance: The server runs the Guarding Circle algorithm on the updated digital twin states to predict and identify potential future collisions.
6. Actuation: If a potential collision is detected, the server issues a control command (e.g., DecelerateOrStop) to the appropriate robot(s) to prevent the collision.

This process repeats continuously, forming a fast, closed-loop control system that is both predictive and responsive.

### 4.2. Edge-Based Feature Extraction

**Figure 4:**
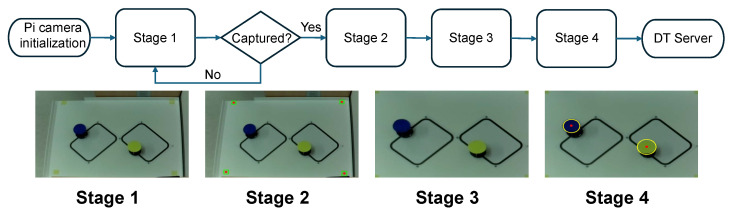
The vision pipeline inside the smart observer: raw frame acquisition, color segmentation, blob filtering, and centroid extraction to identify robots.

The smart observer is a critical component responsible for converting high-dimensional sensor data into lightweight, semantic features. As shown in Figure 4, our implementation uses a computer vision pipeline for this task:

1. Frame Acquisition: Frame acquisition is initiated using the Raspberry Pi Camera V2.1, operating at a resolution of 640 × 480 pixels and a frame rate of 30 FPS. The image acquisition is handled using imutils.VideoStream, which wraps the PiCamera interface for seamless and asynchronous frame grabbing. This choice optimizes for real-time operation while maintaining adequate image quality for low-level feature analysis.
  This raw frame represents a dense, high-dimensional RGB signal I(x,y,c), where $x,y\in Z^{+}$ are pixel coordinates and c∈{R,G,B} denotes the color channel.
2. Color Segmentation: To achieve lighting-invariant object detection, the RGB image is first converted to the HSV color space. HSV separates chromatic content (Hue) from intensity (Value), which provides robustness against illumination changes and shadows. Gaussian blurring with a kernel size of 11×11 is first applied to suppress high-frequency noise:
  (5) $$\begin{array}{c} I_{\mathrm{blurred}}\left(x,y\right)=G\left(x,y\right)*I\left(x,y\right), \end{array}$$
  where G(x,y) is the Gaussian kernel, and ∗ denotes convolution. A color mask is then applied using an empirically calibrated range [lower_bound,upper_bound] in HSV space. This generates a binary image M(x,y)∈{0,255} where white pixels represent regions of interest (ROIs).
3. Blob Analysis: The binary mask often contains noise and fragmented components. To refine this, two morphological operations are applied:
  - Erosion with a 3×3 kernel: removes small isolated noise.
  - Dilation with a 3×3 kernel: restores the size of significant regions.
  Mathematically, erosion ε and dilation δ are defined as follows:
  (6) $$\begin{array}{c} \varepsilon \left(M\right)\left(x,y\right)=\min_{(i,j)\in K}M\left(x+i,y+j\right), \end{array}$$
  (7) $$\begin{array}{c} \delta \left(M\right)\left(x,y\right)=\max_{(i,j)\in K}M\left(x+i,y+j\right), \end{array}$$
  where *K* is the structuring element (kernel). Contours in the filtered mask are then extracted using OpenCV’s findContours(). This yields a set $C=\{C_{1},C_{2},\ldots ,C_{n}\}$ of external contours, each representing a detected object or robot marker.
4. Centroid Extraction: Each contour $C_{i}$ is a set of boundary pixels. To compute its geometric center (centroid), image moments are used. For a binary shape *S*, the spatial moment of order (p,q) is defined as follows:
  (8) $$\begin{array}{c} M_{pq}=\sum_{x}\sum_{y}x^{p}y^{q}I\left(x,y\right). \end{array}$$
  For centroid computation:
  - $M_{00}$: area of the blob.
  - $M_{10},M_{01}$: first-order spatial moments.
  The centroid $\left(x_{c},y_{c}\right)$ is then derived as follows:
  (9) $$\begin{array}{c} x_{c}=\frac{M_{10}}{M_{00}},\;y_{c}=\frac{M_{01}}{M_{00}}. \end{array}$$
  This computation is implemented as follows: Contours with very small areas (e.g., noise) are filtered out by imposing a minimum radius threshold (e.g., r>10 pixels). Each valid detection is annotated and appended to the output structure.

**Figure 5:**
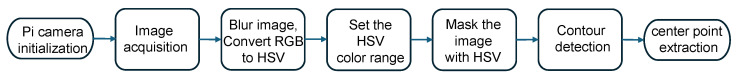
Conversion of pixel centroids from the image to real-world arena coordinates via a homography transformation. These (x,y) coordinates are sent as feature messages to the DT server.

Finally, as shown in Figure 5, a pre-calibrated homography transformation is applied to map these pixel centroids to real-world coordinates (x,y) within the operational arena. These coordinate pairs are then packed into a small data packet for transmission.

### 4.3. Guarding Circle Collision Avoidance Algorithm

Once the digital twins are updated with the latest state information, the DT server executes the Guarding Circle algorithm as the core safety mechanism for inter-robot collision avoidance. The Guarding Circle algorithm was selected due to its computational efficiency, which is critical for meeting the strict real-time demands of the E-DTNCS. In a system where end-to-end latency must be minimized, the collision avoidance strategy must be computationally tractable. While more sophisticated alternatives, such as Model Predictive Control (MPC) or Velocity Obstacles (VO), can provide more optimal trajectories, they often incur a higher computational load that could introduce unacceptable delays. The Guarding Circle approach provides a conservative yet analytically sound mechanism for ensuring safety without compromising the real-time responsiveness of the control loop.

The core idea is to define a circular safety zone, or *guarding circle*, of a predefined radius $r_{g}$ around the center of each robot’s digital twin. A potential collision is predicted if the guarding circles of any two robots, say robot *i* and robot *j*, overlap. This condition is checked by comparing the Euclidean distance between their centers, $d_{ij}$, with the sum of their radii ($2r_{g}$ for identical circles).

(10) $$\begin{array}{c} \mathrm{Potential}\;\mathrm{Collision}\;\mathrm{if}\;d_{ij}\leq 2r_{g}. \end{array}$$

The radius $r_{g}$ is chosen during an offline configuration stage. It must be large enough to account for the physical size of the robot plus a safety margin that considers factors like the robot’s maximum speed and braking distance, and the system’s end-to-end latency. This ensures that any issued avoidance command is executable within the robot’s physical performance limits.

The complete algorithm, which runs on the DT server, is detailed in Algorithm 1. It systematically checks all unique pairs of robots for potential collisions and resolves conflicts based on a simple priority rule. This priority-based logic, while simple, is computationally lightweight and ensures a deterministic decision can be made with minimal processing delay. This is critical for adhering to the system’s overall low-latency requirements. In our implementation, when a conflict is detected between two robots, the one with the higher current velocity is commanded to decelerate or stop, allowing the slower one to proceed. This logic prevents deadlocks and ensures smooth traffic flow. The resulting control commands are then transmitted to the physical robots for execution.
**Algorithm 1** Guarding-Circle Collision Avoidance.**Require:** Set of robots R={1,…,N};  1:Latest pose vector $p_{i}=\left(x_{i},y_{i},\theta_{i},v_{i}\right)$ for each i∈R;  2:Guarding circle radius $r_{g}$;**Ensure:** Control command set $U=\{u_{1},\ldots ,u_{N}\}$;     **Stage 0 - Offline Configuration**  3:Choose $r_{g}$ based on worst-case stopping distance, robot dimensions, and safety regulations.     **Stage 1 - Digital Twin Update**  4:Receive feature packet from the edge observer and update the state of the digital twins ${\hat{p}}_{i}$ for all i∈R.     **Stage 2 - Overlap Detection**  5:Initialize A←∅                                                  ▹ Set of potentially colliding pairs  6:**for all **i∈R** do**  7:    **for all** j∈R** with **j>i **do**  8:        Calculate distance $d_{ij}\leftarrow \sqrt{{\left(x_{i}-x_{j}\right)}^{2}+{\left(y_{i}-y_{j}\right)}^{2}}$  9:        **if** $d_{ij}\leq 2r_{g}$ **then**10:           Add pair to conflict list: A←A∪{(i,j)}11:        **end if**12:    **end for**13:**end for**     **Stage 3 - Conflict Resolution**14:Initialize all commands: $u_{k}\leftarrow \mathrm{Proceed}$ for all k∈R.15:**for all **(i,j)∈A** do**16:    **if** $v_{i}>v_{j}$** then**                                   ▹ Example priority rule: faster robot yields17:        $u_{i}\leftarrow$ DecelerateOrStop18:    **else**19:        $u_{j}\leftarrow$ DecelerateOrStop20:    **end if**21:**end for**     **Stage 4 - Actuation**22:Transmit the final control command set U to the physical robots.

## 5. Experimental Setup

### 5.1. Experimental Environment

To empirically evaluate the efficacy and operational performance of the proposed E-DTNCS, we established a comprehensive testbed environment. The testbed replicates a controlled indoor robotic navigation scenario, allowing for real-time collision detection, environmental reconstruction, and DT-based command execution. The testbed utilizes a mobile robot platform, specifically a remote-controlled vehicle developed by Waveshare Electronics, which is integrated with a Raspberry Pi 3B+ as its processing unit. Detailed hardware specifications of the Raspberry Pi 3B+ are enumerated in Table 1. The robot is equipped with a dual-wheel differential drive system, facilitating precise maneuverability and bidirectional movement along a predefined black-line trajectory embedded within the test environment. The path-following capability is implemented using an onboard infrared (IR) sensor array, which continuously monitors reflectivity contrasts to detect and follow the black-line path. The IR sensors output values indicative of the robot’s relative position on the line, enabling real-time correction of its course.

All experiments were carried out on a dedicated IEEE 802.11n WLAN in the 2.4 GHz band. The access point was configured for 40 MHz with transmit power set to the 20 dBm EIRP (100 mW). Both RTS/CTS and MAC-layer fragmentation were effectively disabled by setting the RTS threshold to 2347 bytes and the fragmentation threshold to 2346 bytes, so that no additional control frames were inserted for the short feature packets used in the edge-enabled link. The beacon interval remained at the default 100 ms; at this setting, beacon traffic occupies well under one percent of the channel’s duty cycle and does not affect the measured latency. When the robot wirelessly receives control commands from the DT server, it forwards them to the motor driver through the Raspberry Pi’s general-purpose input/output (GPIO) pins. The driver then executes the commands, producing the required motion and completing the perception–decision–action loop.

To support real-time sensing and DT updates, an edge-side smart observer module is installed at the perimeter of the test arena. The module consists of a single board computer with an integrated camera and Wi-Fi interface. The camera streams 640 × 480 video, supplying a continuous view of the robot workspace. Each frame is forwarded over the local WLAN to the DT server, where feature extraction routines estimate the robot’s location, orientation, and context within the physical space. These features drive real-time updates of DT, keeping the simulated environment synchronized with its physical counterpart. The DT server runs on a workstation equipped with a 3.4 GHz Intel Core i7 9700K CPU and 128 GB of DDR4 RAM. The DT server applies the guarding-circle algorithm to predict trajectory overlaps and indicates imminent collisions. When a potential collision is predicted, the server issues an avoidance command over the wireless link, such as stopping, slowing down, or changing the route. The robot executes the instruction immediately, eliminating the hazard and closing the perception–decision–action loop. We consider two principal categories of collision risks commonly encountered in multi-robot systems: intersection collision and rear-end collision.

#### 5.1.1. Intersection Collision

**Figure 6:**
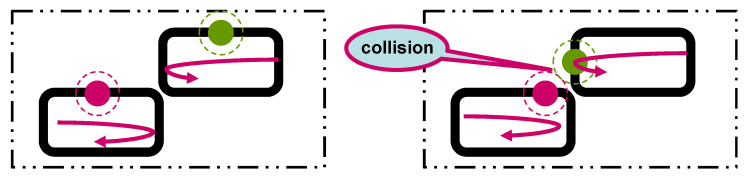
Intersection collision scenario.

This occurs when two mobile robots operate along distinct paths that converge or overlap at certain critical points. At the intersection, both robots may arrive simultaneously, leading to a potential collision if no coordination or control intervention is implemented. Figure 6 illustrates this scenario, highlighting the convergence point and the risk zone where temporal path overlap may result in a collision.

#### 5.1.2. Rear-End Collision (Loop Collision)

**Figure 7:**
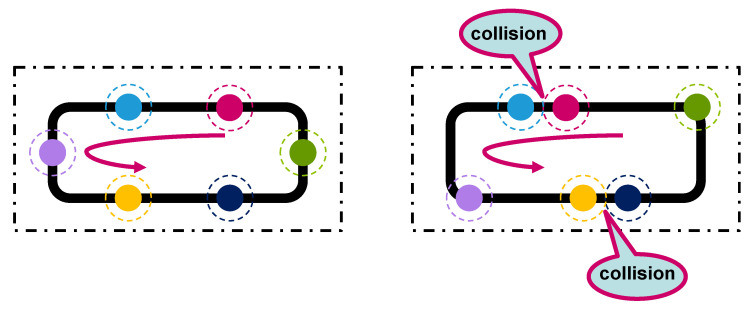
Rear-end collision (loop collision) scenario.

In this scenario, multiple robots navigate a shared contour path, each maintaining its own speed, so they pass any given point on the loop at different times. Unlike intersection collisions, rear-end collisions arise from the inability of trailing robots to maintain a safe distance from the robots ahead. This is a common situation in path-following systems where multiple agents share a single trajectory. Figure 7 depicts this configuration, where delayed deceleration or inaccurate spacing can cause one robot to collide into the rear of another.

### 5.2. Experimental Procedure

The evaluation proceeds as follows. The robots are placed on pre-defined tracks with different intersection layouts. The smart observer streams position features to the DT server, which in turn issues motion commands. The smart observer executes a four-step vision pipeline illustrated in Figure 4. First, a raw frame is captured. Second, simple color segmentation separates the dark line and the bright robot bodies from the background. Third, small artifacts are removed, and each robot blob is labeled. Fourth, the pixel centroid of every blob is calculated. Figure 5 shows the next stage: these centroids are mapped to arena coordinates by a planar homography and packed into compact (x,y) feature messages that travel to the DT server.

Each incoming feature packet updates the virtual replica, after which the guarding-circle routine checks for overlapping safety zones. When an overlap is detected, the server sends an avoidance back to the appropriate robot. Because only a handful of bytes leave the observer on every cycle, the end-to-end delay stays well below the safety limit.

**Figure 8:**
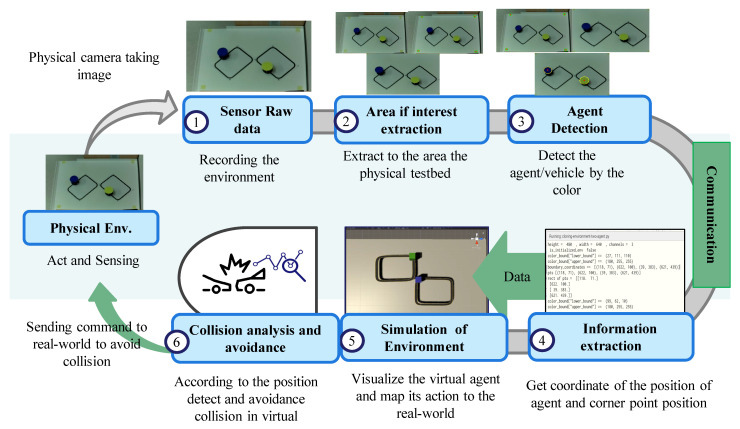
End-to-end data flow in the experimental setup.

The complete data path, from camera capture to motor actuation, is summarized in Figure 8. The physical robots, smart observer, and DT server form a closed loop in which sensing, feature extraction, twin simulation, collision prediction, and actuation occur continuously.

The following operating conditions are tested:

1. Four nominal speeds (0.13, 0.14, 0.16, 0.17 m/s);
2. Two traffic patterns—simple loop and intersection.

For each condition, we record (i) the end-to-end latency, (ii) the number of avoidance commands issued, and (iii) the observed collision count.

These performance metrics enable a direct and meaningful comparison between the E-DTNCS-based pipeline, which transmits compressed semantic feature packets, and the conventional DT-based pipeline, which relies on raw image transmission. Additionally, they provide a benchmark against a baseline approach that lacks integration of a Digital Twin model for predictive collision avoidance.

## 6. Results and Discussion

### 6.1. Collision Avoidance Comparison

To evaluate the effectiveness of the proposed collision avoidance solution, two experimental setups are considered: intersection collisions (Figure 6) and rear-end collisions (Figure 7). In the intersection collision scenario, two autonomous mobile robots follow independent trajectories that converge at a shared intersection, as illustrated in Figure 6. A potential collision arises when both robots approach the intersection simultaneously.

To validate the effectiveness of the proposed E-DTNCS, we conducted a series of experiments under four distinct velocity profiles, simulating various real-world operational conditions. For each velocity profile, we compared the collision rate between a baseline setup without DT and one incorporating the DT framework. It is important to note that for the ’without-DT’ baseline configuration, all onboard reactive control mechanisms (e.g., proximity sensor-based avoidance) were intentionally deactivated. This was performed to establish a true baseline and enable a fair, controlled comparison that isolates the performance gains attributable solely to the centralized DT and E-DT control architectures.

The collision rate was used as a key performance metric to quantify the system’s robustness in avoiding inter-robot collisions under dynamic conditions. The collision rate was computed through a controlled empirical procedure as follows: for each velocity profile, the autonomous mobile robots were programmed to follow a predefined trajectory repeatedly for 20 trials, ensuring consistent and repeatable motion behavior. During each trial, the observer recorded logs indicating whether a collision occurred.

The total number of collisions observed over the 20 repetitions was denoted as $C_{v}$, where *v* represents the specific velocity profile. The collision rate $R_{c}$ for that profile was then calculated using the following formula:

(11) $$R_{c}=\frac{C_{v}}{N},$$

where N=20 is the total number of trajectory executions under that profile. This yields a normalized collision rate, representing the likelihood of a collision occurring per trial. Lower values of $R_{c}$ indicate a more reliable collision avoidance capability. By comparing the computed $R_{c}$ values for both the baseline and DT-enabled systems across all velocity profiles, we were able to quantitatively assess the contribution of the digital twin framework to collision mitigation under different dynamic regimes.

**Figure 9:**
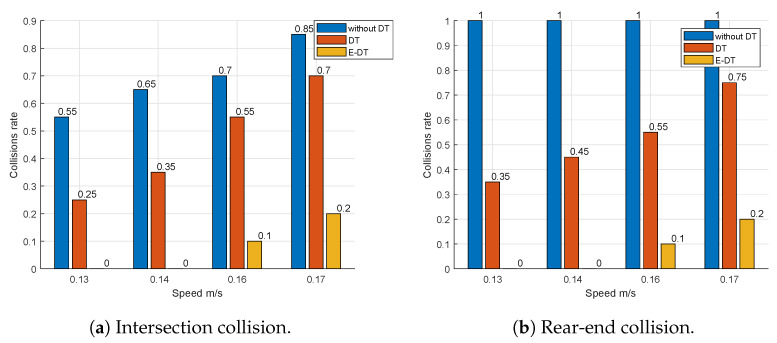
The collision rate as a function of speed of mobile robots.

The results, presented in Figure 9a, clearly demonstrate that the DT-enabled system significantly outperforms the non-DT counterpart. At every tested speed, the *without-DT* baseline—robots run with no central coordination—shows the highest collision ratio (55–85%), emphasizing the risk of leaving the fleet unmanaged. Streaming raw camera frames to the server (*DT*) roughly halves that risk, but collisions still occur because the added transmission latency delays the guarding-circle response. With *E-DT*, the uplink is reduced to compact feature packets; as a result, the collision rate drops below 20% and is often zero, demonstrating that low-latency updates are essential for safe operation.

The second experimental scenario extends the investigation to a dynamic multi-robot setting, where several robots navigate along a predefined circular or polygonal path, as illustrated in Figure 7. In this setup, the primary challenge shifts from intersection-based conflicts to maintaining safe spacing and avoiding collisions between adjacent robots, particularly under conditions of speed variation and asynchronous motion. Mirroring the methodology used in the intersection collision experiments, we evaluated the performance of the proposed DT framework against a baseline system without DT integration. The focus remained on quantifying the collision rate across different velocity profiles. The results, shown in Figure 9b, further reinforce the effectiveness of the DT approach. The same trend appears in the rear-end scenario: anarchic motion (*without-DT*) produces near-certain rear-end contact at moderate speeds, while raw-image DT cuts the rate but cannot eliminate it. The edge-enabled DT (*E-DT*) again performs best; collisions are prevented in most runs, even when speeds approach 0.17 m/s. Specifically, the *E-DT* system consistently exhibited a lower collision rate, attributable to its ability to minimize latency and enable faster, predictive coordination between robots.

**Figure 10:**
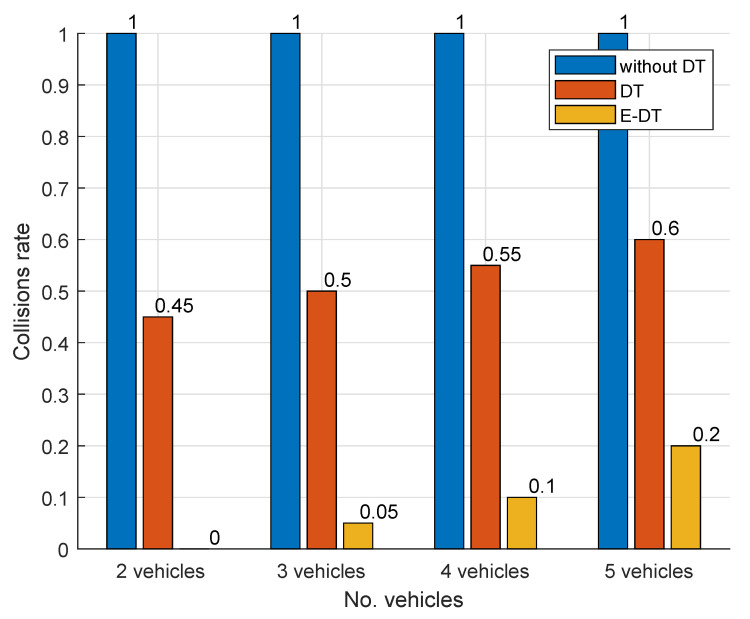
The collision rate as a function of the number of mobile robots.

Additional experiments were performed to assess the impact of robot density on collision risk. As shown in Figure 10, when the fleet grows from two to five robots, an unmanaged baseline (*without-DT*) collides in every run, giving a constant collision rate of 1. Using DT with raw images (*DT*) keeps the rate near 0.45 for two robots, but it rises to roughly 0.60 as density increases, showing that the network latency becomes more harmful in crowded conditions. The *E-DT* maintains the lowest collision rate across all fleet sizes—0 for two robots and 0.20 for five—but the upward trend reveals that even feature-based updates demand tighter timing as the workspace becomes busier. As in previous figures, the *without-DT* curve serves as a worst-case reference, clarifying the safety gains achieved first by DT control and then by its edge-accelerated version.

### 6.2. Inter-Robot Distance Stability Under Varying Fleet Sizes

In multi-robot systems, ensuring a safe inter-robot distance is a fundamental requirement for collision avoidance and coordinated behavior, especially under time delays and dynamic conditions. As robot fleets grow in number and complexity, maintaining consistent spatial separation becomes increasingly challenging due to interaction effects and potential communication latency. To demonstrate this, we analyze how our system manages spacing between robots over time in two configurations—one with a small fleet (two robots) and another with a larger fleet (four robots). This experiment aims to validate the system’s robustness in preserving safety limits and its scalability to larger formations.

**Figure 11:**
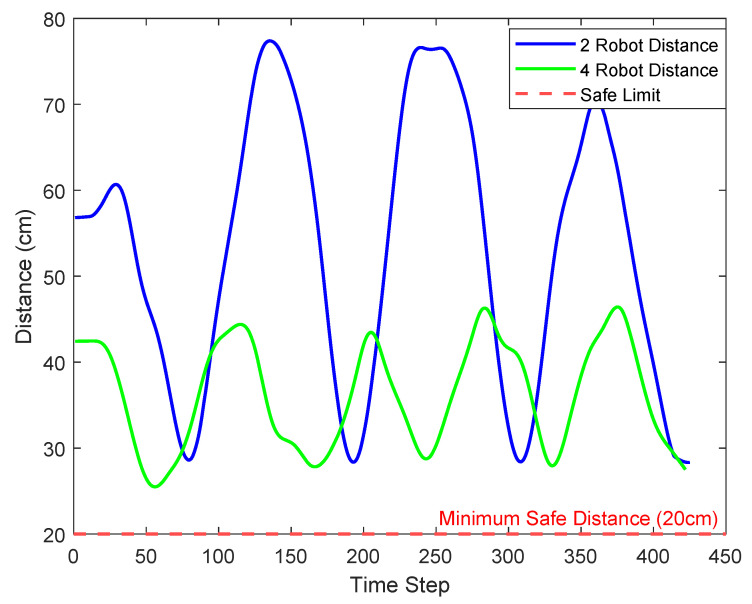
Distance between robots over time for two scenarios: two robots (blue) and four robots (green).

Figure 11 illustrates the time evolution of the inter-robot distances under two deployment scenarios: two robots and four robots. The x-axis represents discrete time steps, while the y-axis shows the measured distance between adjacent robots in centimeters. The red dashed line indicates the minimum safety limit ($d_{\min}=20$ cm), below which collision risk becomes significant. In the case of two robots, the inter-robot distance oscillates with relatively high amplitude, maintaining ample spacing and never breaching the safety threshold. This demonstrates that the control system effectively preserves distance even in sparse configurations.

As the number of robots increases to four, the available spacing is naturally reduced, leading to a tighter formation. However, the green curve confirms that even in this more constrained setup, the minimum safe distance is consistently maintained throughout the simulation. These results highlight the adaptability of the proposed control algorithm: despite the dynamic and potentially destabilizing effects of increased robot density, the system ensures that inter-robot distances remain above the critical safety margin.

This behavior aligns with the theoretical expectation derived from Section 3.3.2. When the end-to-end delay is bounded by $t_{e2e}<\frac{d_{\min}}{v_{\max}}$, the error in inter-robot distance $e_{d}\left(t\right)$ remains non-negative, preventing collisions. Even if a transient deviation occurs during the delay window, the system ensures exponential decay of the error afterward. Hence, the empirical results provide both practical validation and theoretical grounding for the stability and safety of the proposed method in managing dynamic multi-robot formations.

### 6.3. Communication Efficiency

Efficient and low-latency communication is a fundamental requirement for the successful deployment of E-DTNCS in multi-robot collision avoidance and remote control applications. Minimizing transmitted data while preserving real-time update capability is critical, as excessive communication overhead can significantly increase latency, thereby degrading system responsiveness and decision-making accuracy. In this study, we investigated the role of edge computing-based smart observers in enhancing communication efficiency by reducing the size of transmitted information. Specifically, we compared a conventional DTNCS pipeline, which transmits raw image data, with the proposed E-DTNCS framework that communicates compact semantic feature vectors. The evaluation was performed across varying fleet sizes from one to four autonomous robots to assess scalability and performance under increasing communication demands.

**Figure 12:**
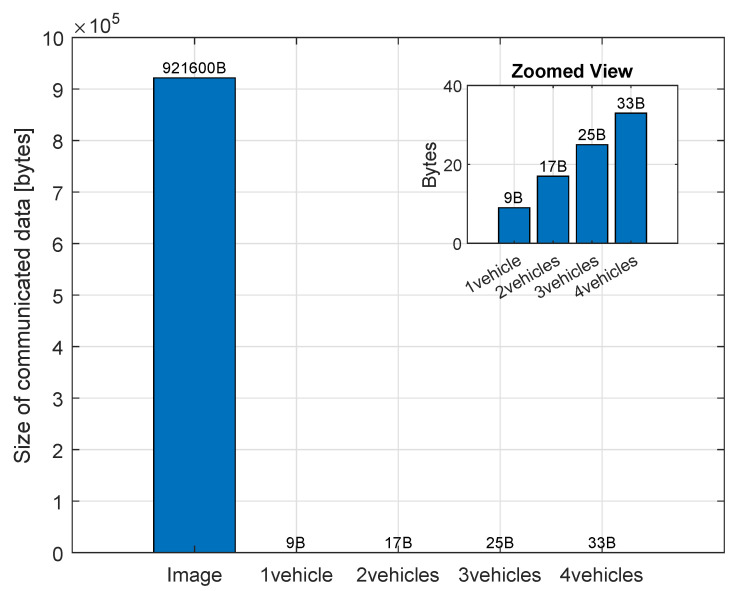
Comparison of transmitted data size between conventional DTNCS (raw image transmission) and the proposed E-DTNCS (semantic feature transmission) across different fleet sizes (1–4 robots).

Experimental results revealed a 99.99902% reduction in data transmission volume when employing the smart observer as indicated in Figure 12. Specifically, the raw-image observer transmitted 921,600 bytes per frame, while the smart observer reduced this to a mere 9 bytes by extracting and sending only the essential coordinate data of autonomous robots. This drastic reduction in data volume alleviates network congestion, allowing multiple robots to communicate with the DT server without overwhelming network resources.

**Figure 13:**
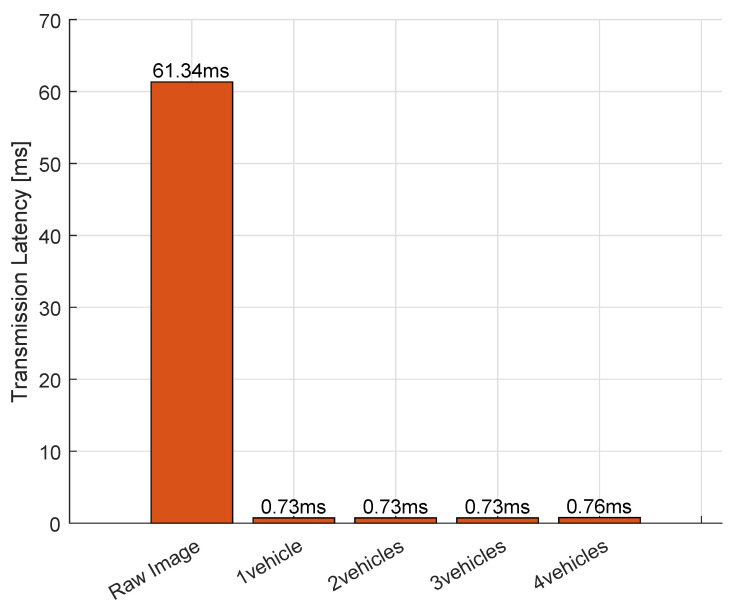
Comparison of transmission latency between conventional DTNCS (raw image transmission) and the proposed E-DTNCS (semantic feature transmission) across different fleet sizes (1–4 robots).

Figure 13 demonstrates a 98.8% reduction in transmission latency achieved by our E-DTNCS compared to conventional approaches. The baseline system, which relies on raw image data transmission from the puppet observer to the DT server, exhibits a mean latency of 61.34 ms. In contrast, our E-DTNCS framework reduces this to just 0.76 ms by performing edge-level semantic feature extraction and transmitting only 33 B of compressed feature vectors instead of 921,600 B raw image frames. This 27,927:1 data compression ratio directly translates to near real-time synchronization between physical and virtual domains.

To support large fleets, our system employs semantic feature extraction at the edge, significantly reducing communication overhead. Instead of transmitting raw image frames (921,600 bytes), we transmit only the essential positional data of each robot. The total data payload scales linearly with the number of robots, following the model D=1+8n bytes, where *n* is the number of robots. For example, a fleet of 100 robots would generate a total payload of approximately 801 bytes—less than 0.1% of a single raw image frame. This ensures that communication remains efficient and network congestion is avoided, even as fleet size increases substantially.

**Figure 14:**
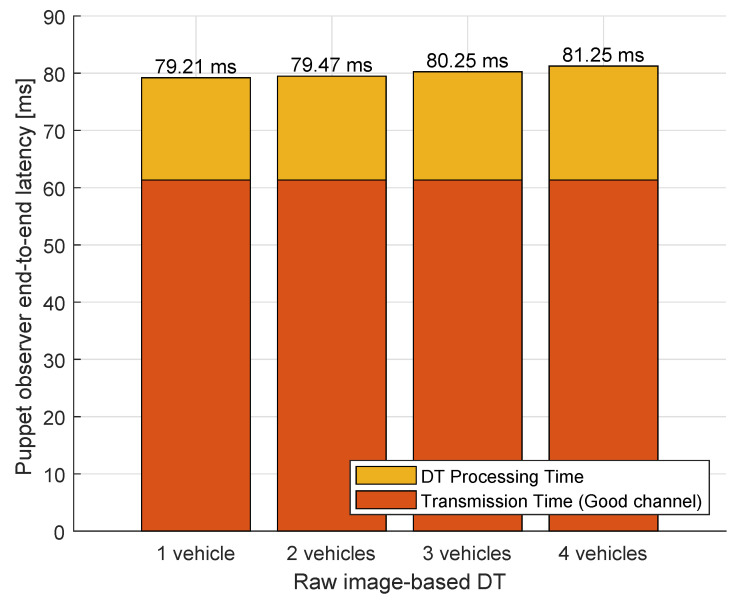
Data processing time in the smart observer.

**Figure 15:**
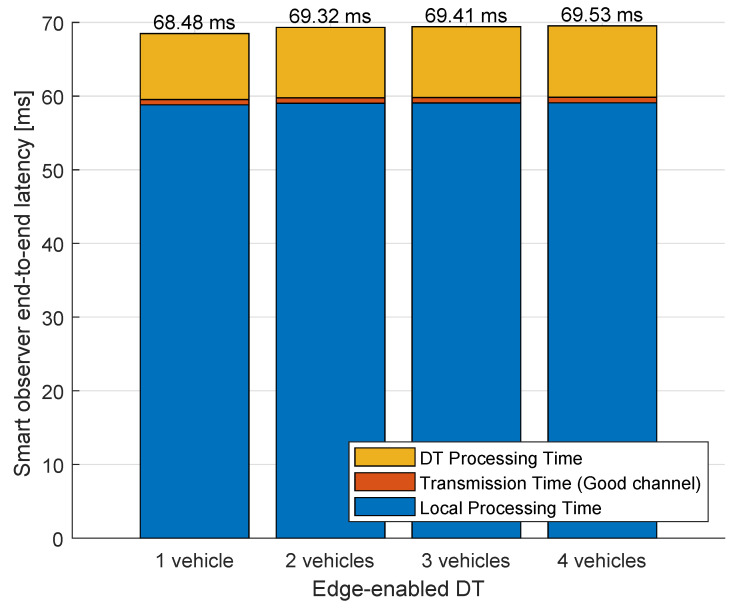
Total delay of E-DTNCS mode.

To assess the effectiveness of our proposed E-DTNCS architecture, we analyzed the end-to-end latency decomposition across two observer configurations: the conventional DTNCS and the proposed E-DTNCS, as illustrated in Figure 14 and Figure 15.

In the DTNCS (puppet observer) setup, raw image frames are transmitted to the DT server for processing. This results in a baseline latency of 79.21 ms in single-robot scenarios, and a non-linear increase to 81.25 ms at four robots (N = 4), where over 89% of total delay is attributed to network transmission time alone. In contrast, the E-DTNCS (smart observer) performs feature extraction locally, transmitting only semantic representations. This edge-based preprocessing yields a reduced initial latency of 68.48 ms a 13.6% improvement over the puppet observer, and exhibits near-constant latency scaling (only +1.05 ms across 1 to 4 robots), with a maximum latency of 69.53 ms at N = 4. Notably, network transmission accounts for just 1.1% of the end-to-end delay in this configuration.

These results highlight the architectural advantage of distributing computational tasks toward the edge. By minimizing reliance on bandwidth-intensive image transmission and avoiding network-induced variability, the E-DTNCS achieves scalable, low-latency performance that remains stable even as the number of robots increases. This design significantly enhances the system’s responsiveness and ensures timely synchronization between the physical and digital domains an essential requirement for real-time multi-robot coordination and collision avoidance.

### 6.4. Validating E-DTNCS Reliability Under Channel Impairments

To assess the robustness of the proposed E-DTNCS under varying wireless conditions, we conducted simulations using the IEEE 802.11be (EHT) physical layer model in MATLAB. A single-antenna transmission configuration was used (1 Tx, 1 Rx) with 320 MHz bandwidth (CBW320), representative of high-throughput edge-to-cloud communication scenarios. Two data payloads were evaluated: a 921,600-byte PSDU for conventional raw image transmission, and a 33 byte PSDU for compressed semantic features used in E-DTNCS. The transmission channel employed a TGax multipath fading model (Model-B) with SNR levels ranging from 36 dB to 51 dB in 5 dB increments. Modulation and coding were fixed at MCS 13 to ensure a fair comparison. For each configuration, up to 100 packets were transmitted per SNR level, with transmission halted early if 10 errors occurred. A complete PHY layer pipeline, including synchronization, channel estimation, and bit recovery, was implemented to ensure realistic performance evaluation. PER results were logged across all trials and visualized on a logarithmic scale to reveal trends under both high-loss and low-error regimes.

**Figure 16:**
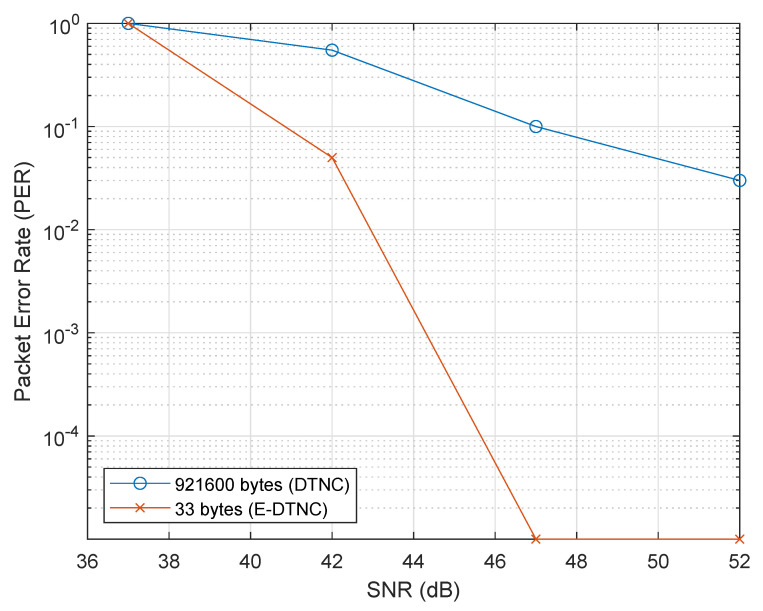
Packet Error Rate (PER) versus SNR for DTNCS (raw image) transmission versus E-DTNCS (semantic feature) transmission.

Figure 16 presents a comparative analysis of the packet error rate (PER) for both the conventional baseline and the E-DTNCS across various SNR values. The results highlight the pronounced impact of payload size on communication reliability. In the baseline approach, where raw image frames (921,600 bytes) are transmitted, the PER remains critically high at low SNR values. Specifically, at 37 dB, all packets are lost (PER = 1.0), and even at 52 dB, the PER only drops to 0.03, indicating persistent vulnerability to channel noise. This sensitivity is attributed to the large payload, which increases the likelihood of bit errors accumulating across the frame.

In contrast, the proposed E-DTNCS, which only transmits 33 bytes of semantic features, exhibits far superior robustness. The PER falls to 0.05 at just 42 dB and reaches 0 at SNRs of 47 dB and above. Notably, even at 42 dB, a level at which the baseline still experiences a 55% error rate, E-DTNCS maintains over 95% reliability. This demonstrates that smaller, semantically rich packets are significantly less susceptible to degradation, resulting in fewer retransmissions and better temporal consistency in the control loop.

From a statistical standpoint, the average PER for the baseline system across the SNR range is 0.35, compared to just 0.01 for E-DTNCS, making it 35 times more reliable. Furthermore, the baseline still suffers losses at high SNRs, achieving only a 90% success rate at 47 dB, while the E-DTNCS consistently delivers 100% success beyond that point. These results validate the core hypothesis: by drastically reducing the payload size through semantic feature extraction, E-DTNCS ensures reliable packet delivery under channel conditions that would otherwise cause failure in conventional systems.

This reliability has significant implications for multi-robot systems where control commands must arrive within strict deadlines. The reduced PER leads to fewer retransmissions, which not only conserves energy at the edge, but also stabilizes the control loop timing—an essential factor for collision avoidance. In sum, the E-DTNCS outperforms traditional approaches by maintaining high communication fidelity even under sub-optimal SNR conditions, confirming its suitability for safety-critical robotic applications.

**Figure 17:**
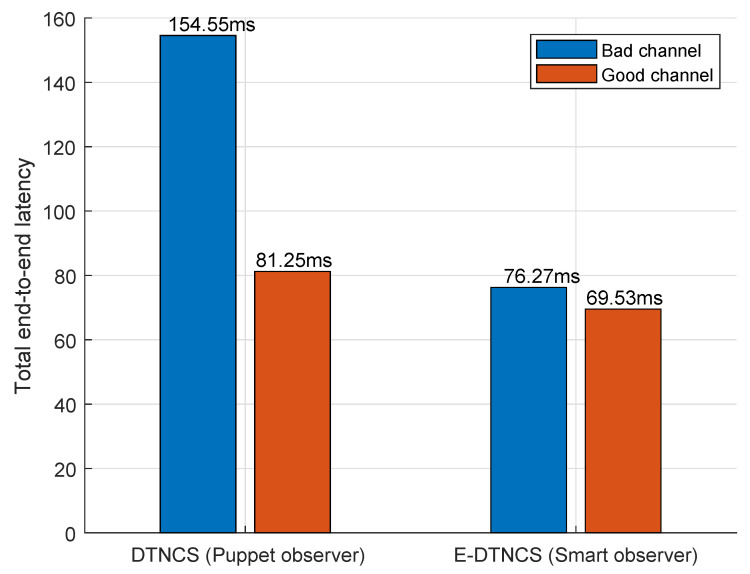
Comparison of total end-to-end latency between DTNCS (puppet observer) and E-DTNCS (smart observer) under good and bad wireless channel conditions.

To further evaluate the robustness of the proposed E-DTNCS architecture, we compared its end-to-end latency performance under varying wireless channel conditions. Figure 17 presents the measured total latency for both DTNCS and E-DTNCS configurations under good and bad channel conditions. In the DTNCS system, which transmits full raw image frames, latency is highly sensitive to channel degradation. Under poor SNR conditions, the end-to-end latency spikes to 154.55 ms—nearly double the latency in a good channel (81.25 ms). This large discrepancy is primarily due to increased packet loss and retransmissions associated with the large 921,600-byte payload, which overwhelms the channel and introduces significant transmission delay.

In contrast, the E-DTNCS system transmits only 33 byte semantic feature packets. Even under poor channel conditions, the latency only rises modestly to 76.27 ms, representing a negligible increase of less than 10% compared to its good-channel performance (69.53 ms). This resilience is attributed to the drastically reduced payload size, which not only minimizes the chance of bit errors but also decreases the burden on the network stack, thereby maintaining low latency and consistent performance.

These results reinforce the design strength of the proposed E-DTNCS: its edge-based semantic compression enables stable and efficient operation even under unfavorable network conditions. Most notably, the E-DTNCS under a bad channel (76.27 ms) still outperforms the conventional DTNCS under a good channel (81.25 ms), clearly demonstrating its superiority in robustness and communication efficiency. This characteristic is critical for safety-critical applications such as multi-robot coordination and collision avoidance, where maintaining low and predictable latency is essential for timely decision-making and system reliability.

## 7. Limitations and Future Work

The proposed E-DTNCS architecture has shown strong performance in controlled indoor environments using predefined trajectories, enabling focused evaluation of core functionalities. However, real-world deployment requires scalability to dynamic, unstructured settings. Future research will thus extend validation to outdoor environments with unpredictable obstacles and environmental variability.

The current vision-based pipeline, which employs color segmentation, performs well under stable conditions but is sensitive to lighting changes and occlusions. To improve robustness, future iterations will incorporate deep learning-based perception methods, such as CNNs and transformer models, to support reliable operation in complex scenarios.

Moreover, the semantic data stream currently includes only positional coordinates to minimize bandwidth usage, omitting dynamic states like orientation and velocity. While sufficient for line-following tasks, richer state representations will be required for free-space navigation and predictive control in cooperative multi-agent systems.

Finally, the collision avoidance strategy is tailored to robot–robot interactions under predictable dynamics. Upcoming work will generalize this to include dynamic, heterogeneous agents, leveraging reinforcement learning for adaptive control and exploring 5G/6G technologies to ensure low-latency, high-reliability communication in real-world deployments.

## 8. Conclusions

This article presented a comprehensive Edge-Enabled Digital Twin networked control system (E-DTNCS) designed for low-latency and efficient coordination in multi-robot environments. Central to our architecture is the use of edge-based smart observers that extract semantic features at the source, significantly reducing the size of transmitted data. By shifting heavy computation and perception tasks to the edge, the system achieves drastic improvements in communication efficiency and responsiveness. Our results demonstrate a 99.996% reduction in transmitted data compared to traditional raw image-based pipelines. This reduction translates to lower network congestion and faster decision-making, even under increasing fleet sizes. Additionally, the system maintains stable latency, with only minimal increases as more robots are added, highlighting its scalability, with only 1.1% of end-to-end latency attributed to network transmission. Beyond communication performance, the E-DTNCS also proves effective in maintaining safe inter-robot distances. Experimental evaluations show that the system preserves the minimum safe separation across various robot counts, validating its reliability for real-time collision avoidance. Notably, even under degraded wireless channel conditions, E-DTNCS outperforms conventional methods operating under ideal conditions, due to its robustness and low packet error rate. However, the system assumes stable edge-to-cloud connectivity and may require backup mechanisms under complete network failures.

## Figures and Tables

**Table 1:** Hardware specifications of Raspberry Pi 3B+.

Specification	Value
SOC	Broadcom BCM2837B0
Core Type	Cortex-A53 64-bit
No. of Cores	4
GPU	VideoCore IV
CPU Clock Speed	1.4 GHz
RAM	1 GB LPDDR2
Power Requirements	1.13 A @ 5 V

## Data Availability

The raw data supporting the conclusions of this article will be made available by the authors on request.

## Abbreviations

The following abbreviations are used in this manuscript:

CPU	Central Processing Unit
DT	Digital Twin
DTNCS	Digital Twin Networked Control Systems
E-DT	Edge-Enabled Digital Twin
E-DTNCS	Edge-Enabled Digital Twin Networked Control System
GB	Gigabyte
GPIO	General Purpose Input/Output
IR	Infrared
LiDAR	Light Detection and Ranging
PER	Packet Error Rate
SNR	Signal-to-Noise Ratio
UDP	User Datagram Protocol
WLAN	Wireless Local Area Network
